# Specific detection of fungal pathogens by 18S rRNA gene PCR in microbial keratitis

**DOI:** 10.1186/1471-2415-8-7

**Published:** 2008-04-29

**Authors:** Zunaina Embong, Wan Hazabbah Wan Hitam, Chan Yean Yean, Nur Haslindawaty Abdul Rashid, Balqis Kamarudin, Siti Khaironi Zainal Abidin, Sabariah Osman, Zainul F Zainuddin, Manickam Ravichandran

**Affiliations:** 1 Department of Ophthalmology, School of Medical Sciences, Universiti Sains Malaysia, Malaysia; 2 Department of Medical Microbiology and Parasitology, School of Medical Sciences, Universiti Sains Malaysia, Malaysia; 3 School of Health Sciences, Universiti Sains Malaysia, Malaysia; 4 Universiti Malaya, Malaysia; 5 Universiti Teknologi Malaysia, Malaysia

## Abstract

**Background:**

The sensitivity and specificity of 18S rRNA polymerase chain reaction (PCR) in the detection of fungal aetiology of microbial keratitis was determined in thirty patients with clinical diagnosis of microbial keratitis.

**Methods:**

Corneal scrapings from patients were used for Gram stain, culture and PCR analysis. PCR was performed with primer pairs targeted to the 18S rRNA gene. The result of the PCR was compared with conventional culture and Gram staining method. The PCR positive samples were identified by DNA sequencing of the internal transcribed spacer (ITS) region of the rRNA gene. Main outcome measures were sensitivity and specificity of PCR in the detection of fungus in corneal keratitis.

**Results:**

Combination of microscopy and culture gave a positive result in 11 of 30 samples of microbial keratitis. PCR detected 10 of 11 samples that were positive by conventional method. One of the 19 samples that was negative by conventional method was positive by PCR. Statistical analysis revealed that the PCR to have a sensitivity of 90.9% and specificity of 94.7% in the detection of a fungal aetiology in microbial keratitis.

**Conclusion:**

PCR is a rapid, sensitive and useful method to detect fungal aetiology in microbial keratitis.

## Background

Microbial keratitis is a serious ocular infection that can cause corneal scarring and opacification. The principal known causes of microbial keratitis are bacteria and fungi [1-5]. The incidence of fungal keratitis varies across the United States, ranging from less than 1% in New York, 17% in Texas and 35% in Florida [6]. A wide range of fungi have been isolated. Fusarium and Aspergillus are the most common pathogens, representing 40% and 20%, respectively [7]. In Malaysia, only isolated cases of fungal keratitis have been reported; however, in the neighboring country of Singapore, the most commonly identified organisms in keratitis samples were *Fusarium *sp. (52%) and *Aspergillus flavus *(17%) [8,9].

The incidence of fungal keratitis has increased; inadequate therapy and inaccurate or delayed diagnosis have led to generally poor visual outcomes [10,11]. Its diagnosis remains a difficult problem, although the history and clinical appearance may suggest fungal keratitis. Conventional microbiological detection of fungus in clinical samples requires 2 days to one week [12]. Gram stain and culture techniques occasionally fail in the detection of fungal keratitis. The main reason for this phenomenon is the presence of a relatively small number of causative infectious pathogens in the scraping specimens, which in many cases are non-viable for culture [13].

The basis for effective treatment is rapid diagnosis of the disease and identification of its causative agent. The use of the polymerase chain reaction (PCR) offers great advantage compared with conventional microbiological testing [13-16]. Pan-fungal primers specific for the conserved sequence of 18S and 28S rRNA gene common to all fungi have been used to detect fungal pathogen in clinical specimens [17]. The 28S rRNA gene PCR evaluation was carried out by Anand et al. in 2000 [17]. The 18S rRNA gene-based PCR for the detection of airborne fungi has been reported, but this method requires specific oligonucleotide probes and hybridization [18]. The aim of this study is to evaluate a PCR method based on a new set of primers targeted to 18S rRNA gene for rapid detection of pan-fungal aetiology in microbial keratitis.

## Methods

### Reference strains

Various filamentous fungi (*Penicillium *sp, *Fusarium *sp, *Alternaria *sp, *Aspergillus versicolor*, *Aspergillus fumigatus)*, yeast (*Candida albicans*) and bacteria (*Staphylococcus aureus*, *Enterococcus *sp., *Pseudomonas aeruginosa*, *Haemophilus influenzae*) were obtained from the Department of Medical Microbiology and Parasitology, Hospital Universiti Sains Malaysia, Kelantan, Malaysia. Human DNA was also included in the collection.

#### (i) DNA extraction

DNA was extracted from the sample using a commercial Nucleospin DNA extraction kit according to manufacturer's instructions with some modification (Clontech, CA, USA). Briefly, the samples were transferred to a 1.5 ml microcentrifuge tube containing 30 to 50, 0.5 mm diameter glass beads and 0.2% SDS (sodium dodecyl sulfate). Cell destruction was achieved by vortexing the microcentrifuge tube for 15 minutes. Six hundred microliters of sorbitol buffer (1 M sorbitol, 100 mM EDTA, 14 mM β-mercaptoethanol) and 10 μl (200 units) of lyticase (Sigma, Missouri, USA) were added and the solution was incubated at 30°C for 30 minutes. The mixture was then centrifuged at 6000 rpm for 10 minutes. The spheroplast pellet was resuspended in lysis buffer (Nucleospin Kit) and 25 μl of proteinase K (20 mg/ml) was added, mixed by vortexing and incubated at 56°C overnight. The sample was then centrifuged at 10,000 rpm for 5 minutes. The supernatant was transferred to a fresh 1.5 ml microcentrifuge tube following the steps recommended by the kit. The extracted DNA from the standard fungal cultures were quantified spectrophotometrically at OD260/280 nm with ratios between 1.7 to 1.8.

The analytical sensitivity of the primers was evaluated by PCR amplification with serial diluted concentrations (range: 10 ng – 1 fg) of purified genomic DNA isolated from *Aspergillus versicolor *as a representative fungal species.

#### (ii) Primer design

**Figure 1:**
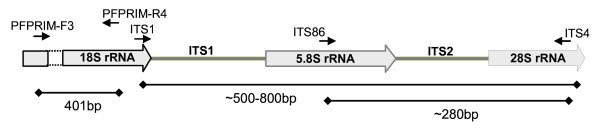
**Schematic representation of the fungal ribosomal 18S rRNA gene and ITS regions with primer binding locations**. ITS- Internal transcribed spacer region.

The 18S rRNA gene was chosen as the target gene for this study. The 18S rRNA gene sequences from *Aspergillus *sp., *Candida *sp. and *Fusarium *sp. were downloaded from GenBank or EMBL databases. A set of unique primers were designed based on the conserved region in all fungal species. To verify the specificity of the designed primers, the BLAST program at the NCBI website was employed to search the primers in short, near exact sequences. Table 1 shows the positions of the primers used in this study with respect to the representative sequences obtained from GenBank (*Aspergillus niger *accession no. D63697, *Fusarium solani *accession no. E17084 and *Candida *sp. accession no. EF120586). Primers were synthesized by First Base Laboratories (Kuala Lumpur, Malaysia). Using ITS1, ITS4 and ITS86 primers, a semi-nested PCR was carried out on the samples that were positive by 18S rRNA PCR [14]. The fungal ribosomal 18S rRNA gene and internal transcribed spacer (ITS) region with primer binding locations are shown in Figure 1.

**Table 1:** The sequences of primers and their relative binding sites on 18S rRNA gene of representative species

Primer name	Sequence 5'............3'	Location (position of base pair)		
		
		*Aspergillus niger *(GenBank accession no. D63697)	*Fusarium solani *(GenBank accession no. E17084)	*Candida *sp. (GenBank accession no. EF120586)
PFPRIM-F3	GACTCAACACGGGGAAACT	1149–1167	1222–1240	1134–1152
PFPRIM-R4	ATTCCTCGTTGAAGAGCA	1522–1539	1605–1622	1509–1526

#### (iii) PCR

PCR reactions for the detection of the fungal keratitis were set up in a clean room with pipettes reserved specifically for this purpose. The PCR reactions were performed in 20 μl volumes containing 2 μl of the genomic DNA sample, 1× PCR buffer containing 750 mM Tris-HCl (pH 8.8 at 25°C), 200 mM (NH_4_)_2 _SO_4_, 0.1% Tween 20; 2.5 mM MgCl_2_; 0.16 mM dNTP Mix; 20 pmol of forward and reverse primers and 0.75 U Taq DNA polymerase (MBI, Fermentas, Lithuania). The mixes were overlaid with 2 drops of mineral oil. Amplification was carried out in a thermal cycler (Eppendorf Mastercycler 5330) with initial denaturation at 95°C for 4 minutes, followed by 30 cycles of denaturation at 95°C for 30 seconds, annealing at 55°C for primer pair PFPRIM-F3 and PFPRIM-R4 for 30 seconds, and extension at 72°C for 30 seconds. The thermal cycles were terminated by a final extension for 5 minutes at 72°C. Purified Aspergillus versicolor genomic DNA served as a positive control and deionised sterile water served as a negative control. The analysis of PCR products were performed on 1% agarose gels. The molecular weight markers Generuler 100 bp and 1 kb DNA ladder (Fermentas, Lithuania) were used and the gel was run at 100 volt for 45 minutes at room temperature. The PCR products were stained with ethidium bromide and visualized by an image analyzer (ChemiImager 5500, Alpha Innotech, CA, USA).

### Clinical study

#### (i) Collection of specimens

Sample size (n = 30) for this cross-sectional study was calculated using Single Proportion Calculator Version 1.0 [19] based on 27% prevalence [20] and 88% assay specificity [21] with 95% confidence interval and 6% precision.

Samples were obtained from patients with microbial keratitis in Hospital Universiti Sains Malaysia and Hospital Kota Bharu for a period of 16 months. Viral keratitis, non-infected corneal ulcer (neutrophic keratitis, chemical burn) and patients less than 12 years old were excluded from the study. Patients' clinical presentation and complications were recorded (Table 2).

**Table 2:** Risk factors, presentation, clinical features and laboratory diagnosis of keratitis

**Patient No**	**Trauma**	**Contact lens**	**Size of lesion (mm)**	**Margin**	**Dry texture**	**Satellite**	**Conj. Inj**	**AC rx**	**Hypopyon**	**Gram stain**	**Culture**	**PCR**	**DNA Sequencing (%)**^**a**^
1	Veg FB	No	8 × 6	Feathery	Yes	No	Yes	Yes	Yes	Hyphae	*Fusarium *sp.	+	*Fusarium solani *(97%)
2	Non-veg FB	No	8 × 8	Feathery	Yes	Yes	Yes	Yes	Yes	Hyphae	*Fusarium *sp.	+	*Cladosporium *sp. (98%)
3	No	No	3 × 4	Feathery	No	Yes	Yes	Yes	Yes	Hyphae	*Fusarium *sp.	+	*Fusarium *sp. (97%)
4	Non-veg FB	No	3 × 2	Feathery	Yes	No	Yes	Yes	Yes	Hyphae	*Fusarium *sp.	+	*Fusarium solani *(97%)
5	No	No	9 × 9	No	No	Yes	Yes	Yes	Yes	Hyphae	*Fusarium *sp.	+	*Fusarium solani *(99%)
6	Veg FB	No	1 × 2	No	No	No	Yes	Yes	No	Hyphae	No growth	+	*Aspergillus flavus *(96%)
7	Non-veg FB	No	1 × 1	No	No	No	Yes	Yes	No	Hyphae	No growth	+	*Trichosporon asahii *(95%)
8	No	No	3 × 5	Feathery	Yes	No	Yes	Yes	No	Hyphae	No growth	+	*Fusarium solani *(87%)
9	No	No	7 × 6	Feathery	Yes	No	Yes	Yes	Yes	Hyphae	*Fusarium *sp.	+	*Glomerella cingulata *(98%)
10	Non-veg FB	No	4 × 5	Feathery	No	Yes	Yes	Yes	No	No organism	*Fusarium *sp.	+	*Fusarium *sp. (97%)
11	Veg FB	No	9 × 9	No	No	No	Yes	Yes	Yes	GNB	*P. aeruginosa*	No	ND
12	No	Yes	2 × 2	No	No	No	Yes	Yes	Yes	GNB	*P. aeruginosa*	No	ND
13	Veg FB	No	6 × 5	No	No	No	Yes	Yes	Yes	GNB	*P. aeruginosa*	No	ND
14	No	Yes	4 × 3	No	No	No	Yes	Yes	Yes	No organism	*P. aeruginosa*	No	ND
15	No	No	10 × 10	Melt	Melt	Melt	Yes	Yes	Yes	No organism	No growth	No	ND
16	Veg FB	No	2 × 3	No	Yes	No	Yes	Yes	Yes	GPC	*S. aureus*	No	ND
17	No	Yes	5 × 4	Feathery	Yes	No	Yes	Yes	Yes	No organism	*P. aeruginosa*	No	ND
18	Non-veg FB	No	2 × 2	No	No	No	Yes	Yes	No	GPC	*S. pneumoniae*	No	ND
19	Non-veg FB	No	8 × 6	Feathery	Yes	No	Yes	Yes	Yes	No organism	No growth	No	ND
20	Veg FB	No	7 × 7	Melt	Melt	Melt	Yes	Yes	Yes	No organism	No growth	No	ND
21	No	Yes	3 × 3	No	No	No	Yes	Yes	No	No organism	*P. aeruginosa*	No	ND
22	No	No	8 × 8	No	No	No	Yes	Yes	Yes	GPC	Group A Streptococci	No	ND
23	Veg FB	No	2 × 1	No	No	No	Yes	Yes	Yes	No organism	No growth	No	ND
24	Non-veg FB	No	2 × 3	Feathery	No	Yes	Yes	Yes	Yes	No organism	No growth	No	ND
25	No	No	1 × 1	No	No	No	Yes	Yes	No	No organism	No growth	No	ND
26	No	No	1 × 1	No	No	No	Yes	Yes	No	No organism	No growth	No	ND
27	Veg FB	No	2 × 2	No	Yes	Yes	Yes	Yes	No	No organism	No growth	No	ND
28	Non-veg FB	No	2 × 3	No	No	No	Yes	Yes	No	No organism	No growth	+	*Fusarium *sp. (97%)
29	No	Yes	2 × 2	No	No	No	Yes	No	No	No organism	*P. aeruginosa*	No	ND
30	Veg FB	No	4 × 2	Feathery	Yes	Yes	Yes	Yes	No	No organism	Fungus NS	No	ND

Veg FB – Vegetative foreign body, Conj. Inj. – Conjunctival injection, AC rx – Anterior chamber reaction, GNB – Gram negative bacillus, GPC – Gram Positive cocci, NS -Non sporulating fungus and ND – Not done.

^a ^Percentage similarity in BLAST analysis of the DNA sequence

Thirty cases of microbial keratitis were investigated in this study. Patients' ages ranged from 14 to 89 years of age; 66.7% were male and 33.3% were female. Clinical features suggestive of fungal keratitis included feathery margin (11 patients, 36.7%), a dry texture (10 patients, 33.3%), and satellite lesions (7 patients, 23.3%). Two patients presented with corneal melting: one patient with graft rejection following penetrating keratoplasty who developed microbial keratitis after exposure to vegetative foreign body and one patient with corneal melting due to post couching (Table 2).

This study was carried out after obtaining the approval from the Ethics committee, School of Medical Sciences, Universiti Sains Malaysia, as per Helsinki declaration. Samples of corneal scraping were obtained from 30 patients (30 eyes) with clinical diagnosis of microbial keratitis. All patients were informed regarding the procedure with written consent. Corneal scrapings were obtained by scraping the edge of the corneal ulcer with a sterile kimura spatula after instillation of topical anaesthesia (0.4% novesin). The procedure was done under a slit lamp biomicroscope. First scraped specimen was stirred in 150 μl of deionised sterile water in a 1.5 ml sterile tube for PCR analysis. Second scraped specimen was cultured onto agar and incubated at 37°C for identification. Third scraped specimen was placed on glass slide for gram staining.

#### (ii) Microscopy and culture

Gram and Giemsa stains were used on representative smears from all clinical specimens and examined under a magnification of 400× and 1000×, respectively. Clinical specimens were cultured at 37°C on blood agar, chocolate agar, Mac Conkey agar, and Thayer Martin agar and at 30°C for Sabouraud's agar under aerobic and anaerobic conditions.

#### (iii) DNA extraction

Corneal tissue genomic DNA was extracted using the commercial Nucleospin DNA extraction kit according to manufacturer's instructions with some modification (Clontech, CA, USA) as described previously. The final volume of the DNA extracted from the corneal samples was 100 μl; 2 μl of this DNA was used for PCR.

#### (iv) 18S rRNA-based PCR assay

The PCR analysis and amplification profile were carried out as described previously. The DNA extracted from patients' samples was tested and the results were compared with conventional diagnostic methods (Table 2).

#### (v) Sequencing of ITS2 region for species identification of PCR detected fungi

Positive samples of 18S rRNA PCR were selected for further species confirmation by DNA sequencing. Primers ITS1, ITS4 and ITS86 were used to amplify the ITS2 region by semi-nested PCR [14]. The PCR products were sequenced using an automated DNA sequencer at Tech Dragon Ltd (Hong Kong, China) and analyzed with the BLAST program provided by the National Center for Biotechnology Information (NCBI) to confirm the fungal species.

## Results

### Primer design

After alignment and visualization of the conserved region, primer pair PFPRIM-F3 and PFPRIM-R4 was selected and used (Table 1).

### Analytical sensitivity and specificity evaluation of 18S rRNA-based PCR assay using reference strains

**Figure 2:**
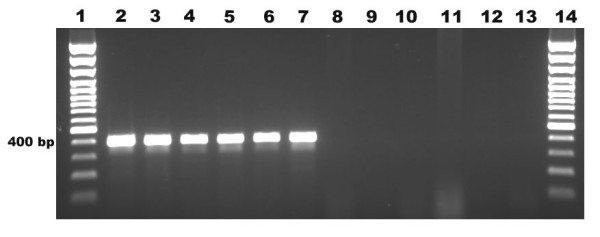
**Specificity of 18S rRNA PCR in yeasts, filamentous fungi and bacterial strains**. Lane 1, 100 bp plus marker; lane 2,*Candida albicans*; lane 3, *Penicillium *sp.; lane 4, *Fusarium *sp.; lane 5, *Alternaria *sp.; lane 6, *Aspergillus versicolor*; lane 7, *Aspergillus fumigatus*; lane 8 *Staphylococcus aureus*; lane 9 *Enterococcus *sp.; lane 10 *Pseudomonas aeruginosa*; lane 11, *Haemophilus influenzae*; lane 12, Human DNA; lane 13, negative control and lane 14, 100 bp plus marker.

**Figure 3:**
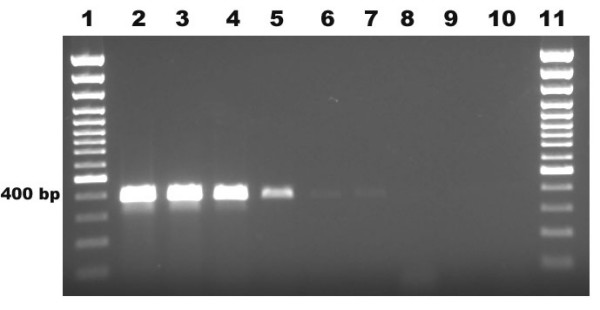
**Sensitivity of PCR at the DNA level using 10-fold dilutions of *Aspergillus versicolor *DNA**. Lane 1, 100 bp plus marker; lane 2, 10 ng; lane 3, 1 ng; lane 4, 100 pg; lane 5, 10 pg; lane 6,1 pg; lane 7, **100 fg; **lane 8, 10 fg; lane 9, 1 fg; lane 10, negative control and lane 11, 100 bp plus marker.

On the basis of the 18S rRNA sequence region, a product of 395 ± 6 bp was amplified by PCR with primer pair PFPRIM-F3 and PFPRIM-R4 from all 6 yeasts and filamentous fungi reference strains of medically important fungal species. None of the 4 bacterial reference strains were positive by PCR amplification (Figure 2). The specificity of the 395 ± 6 bp fragment was verified by DNA sequencing and analysis. No amplification was observed with human DNA and negative control (Figure 2). The sensitivity of the 18S rRNA PCR assay allowed detection of as little as 100 fg of *A. versicolor *genomic DNA by visualization on the agarose gel electrophoresis (Figure 3).

### Diagnostic sensitivity and specificity evaluation of 18S rRNA-based PCR assay using corneal scraping clinical samples

**Figure 4:**
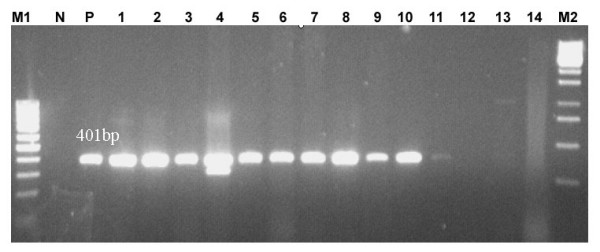
**Detection in clinical samples using 18S rRNA PCR**. Lane M1, 100 bp plus marker; lane N, negative control; lane P, positive control; lane 1–14, clinical samples and lane M2, 1 kb marker.

In the 30 patients with microbial keratitis, a positive fungal aetiology was detected in 11 patients (36.6%) by PCR (Table 2). PCR was considered positive for fungi when there was presence of 395 ± 6 bp PCR product band on agarose gel electrophoresis using PFPRIM-F3/PFPRIM-R4 (Figure 4). Of the 30 patients, conventional Gram stain method yielded positive results in 9 patients (30.0%). 8 patients (26.7%) were detected by the culture method. The most common genus isolated by culture was *Fusarium *sp, accounting for 7 patients (23%). One patient showed growth of non-sporulating fungus (Table 2).

### DNA sequencing of ITS2 region for species identification

The species of the fungus in the PCR positive samples were identified by DNA sequencing of the internal transcribed spacer (ITS) region of the rRNA gene. Segments of 500 to 800 bp of the entire ITS regions, including partial 5.8S rRNA and 28S rRNA, were amplified from the 11 PCR positive samples using one primer pair, ITS1 and ITS4 (Figure 1). The semi-nested ITS2 region PCR of approximately 280 bp was amplified using primer pair, ITS86 and ITS4 (Figure 1). Among the 11 PCR positive samples, *Fusarium *sp. was identified in 7 samples; *Cladosporium *sp., *Aspergillus flavus*, *Trichosporon asahii*, and *Glomerella cingulata *were also identified. Two of the PCR positive samples that were identified as *Fusarium *sp. by culture method were identified as *Glomerella cingulata *and *Cladosporium *by DNA sequencing. This could be due to mixed infection or contamination of the PCR. Combination of microscopy and culture gave a positive result in 11 of 30 samples of microbial keratitis. PCR detected 10 out of 11 samples that were positive by conventional method. One of the 19 samples that were negative by conventional methods was positive by PCR (Table 2).

### Statistical analysis

A statistical analysis revealed that the PCR has a sensitivity of 90.9% and specificity of 94.7% to detect fungal aetiology in microbial keratitis (Table 3).

**Table 3:** Statistical analysis of 18S rRNA PCR diagnostic evaluation

**18S rRNA PCR assay**	**Microscopy and Culture**		**Total**
		
	Positive	Negative	
Positive	10	1	11
Negative	1	18	19
**Total**	11	19	30

^a. ^True positive = 10, false positive = 1, false negative = 1, true negative = 18.

^b. ^This leads to estimates of sensitivity = 0.91 (95% CI = 0.59 to 1.00), specificity = 0.95 (95% CI = 0.74 to 1.00) and a diagnostic odds ratio (DOR) of 180 (7.8 to 8195.8).

^c. ^The positive likelihood ratio (LR+) is 17.3 (95% CI = 2.5 to 117.4) and the negative likelihood ratio (LR-) is 0.10 (95% CI = 0.03 to 0.36).

## Discussion

In this study, PCR was evaluated for its efficacy in the detection of fungal pathogens in corneal scrapings using characterized reference strains. Human genomic DNA was included in the analytical specificity evaluation of the PCR to rule out the non-specific amplification of the patient genomic DNA from the corneal scraping.

In this study 18S rRNA specific primers that amplify medically important fungi were selected and used. The 18S rRNA gene is a multi-copy gene that is slowly evolving and highly conserved among fungi, making it an attractive target for the detection of fungus in clinical specimens. Detection of fungal aetiology by 18S rRNA targeted PCR will be useful in early diagnosis of fungal keratitis and could help in early initiation of antifungal therapy. ITS2 based seminested PCR followed by sequencing was used to identify the species of fungus. ITS has been used to detect fungal pathogens in ocular infections by DNA sequencing and single-stranded conformation polymorphism (SSCP) [14,22].

The current study demonstrates that 18S rRNA-based PCR has high degrees of analytical sensitivity (100 fg) and specificity (100%) for the detection of a wide range of medical significant fungi. Our primers are based on the conserved region of 18S rRNA gene which is designed to detect wide range of fungal strains with the PCR product size of 395 ± 6 bp. However, the ITS based PCR amplifies product size varies between 500 to 800 bp [14]. The difference in product size of ITS primer based PCR often caused difficulty in interpretation the positive from non-specific band in the agarose gel. The non-specific amplification or positive fungal identification can be confirmed only after further sequencing of the ITS PCR product. In addition, ITS primers require two tubes nested PCR method, which is tedious and prone for cross-contamination. The PFPRIM primers based-PCR used in our study is a single tube pan-fungal PCR which can confirm fungal infection. Chen et al. in 2000 have used ITS2 region specific primers to detect fungal species based on PCR product size (ranging from 237 to 429 bp) identification by capillary electrophoresis and restriction polymorphism [23].

The most common initial laboratory procedure done in diagnosis of fungal keratitis is microscopic examination. In this study, a positive microscopic examination was observed in 30% (9) of patients. The percentage of positive stains showing fungal elements was similar to studies in South Florida, in which 33% of Gram stains were positive [20]. Culture yielded positive results in 26.7% of the samples and PCR was positive in 36.6%. The findings of this study agree with those of Lohmann et al. [24] and Anand et al. [17], demonstrating high analytical specificity and sensitivity of the PCR method compared with the conventional method. PCR based test showed improved detection of fungal aetiology in microbial keratitis. Conventionally, culture has been used as 'gold standard' to detect fungal pathogens, but takes 2 days to several weeks for final identification. The main problem with culture from corneal scraping specimens is the small amount of material that can be obtained for diagnosis, increasing the risk of false-negative results. Additionally, some fungal species cannot be cultured or grow slowly and have fastidious growth requirements. This may explain the negative culture results in 3 of the samples used in this study.

The PCR diagnostic test had a sensitivity of 90.9%. PCR detected 10 of 11 samples that were positive by conventional method. One of the samples that were negative by PCR is a case of microbial keratitis due to non-sporulating fungus. This could be due to insufficient fungal elements present in corneal scraping or due to sequence variation of 18S rRNA gene of this fungus. It is possible that different layers of corneal scraping were used for culture, Gram stain and PCR. Hence, the corneal scraping submitted for PCR may have had insufficient fungal elements relative to the samples submitted for culture and Gram stain.

When compared to the Gram stain and PCR, the specificity of PCR was 94.7% with one false positive. However, this sample was found to be positive for *Fusarium *sp. by DNA sequencing. The culture is positive only if the sample contains viable organisms, while a PCR based test will detect both viable and non-viable organisms.

By DNA sequencing of the ITS2 region, we could identify the fungal species [23]. Our results agree with the work of Fitzsimons [7], demonstrating *Fusarium *sp. as the predominated aetiology in ocular fungal keratitis infection. DNA sequencing identified two samples that were misidentified by culture method. Less common organisms, such as *Cladosporium *sp., *Aspergillus flavus*, *Trichosporon asahii *and *Glomerella cingulata*, were identified by DNA sequencing. The role of *Cladosporium *sp., *Trichosporon asahii *and *Glomerella cingulata *in keratitis needs to be investigated.

In most cases of keratitis, the most important laboratory information that the ophthalmologist needs to know is whether the infectious agent is fungal or bacterial. They often hesitate to initiate antifungal therapy in fungal culture negative cases due to the risk of drug associated toxicity. Positive PCR results that are available earlier than culture will justify the use of antifungal agents promptly, resulting in improved visual outcome. This current data agrees with the study by Anand et al. [17] and confirms the efficacy of the PCR assay compared to conventional methods of diagnosis in the clinical setting.

## Conclusion

This study has demonstrated the efficacy of the 18S rRNA gene to detect fungal pathogen in clinical samples. This PCR based test is a rapid, sensitive and a useful method to detect fungal aetiology in microbial keratitis when compared to standard laboratory techniques. Further studies with larger sample size are needed to refine the technique, to calculate sensitivity and specificity, and to establish the value of the technique in managing patients with microbial keratitis.

## Conflict of interest

The authors declare that they have no competing interests.

## Authors' contributions

ZE carried out the molecular genetic and biochemical studies, participated in contribution of conception, design, acquisition of data, analysis and interpretation of data, was involved in drafting of the manuscript, revised it critically for important intellectual content and gave final approval of the version to published. WHWH participated in contribution of conception, design and gave final approval of the version to publish. CYY participated in the design of the study, analysis and interpretation of data; was involved in drafting the manuscript and gave final approval of the version to publish.NH, BK, SKA and SO carried out the molecular genetic and biochemical studies, acquisition of data, analysis and interpretation of data and gave final approval of the version to publish.ZFZ participated in contribution of conception, design, analysis and interpretation of data, was involved in drafting the manuscript and revised it critically for important intellectual content. MR participated in contribution of conception, design, acquisition of data, analysis and interpretation of data, was involved in drafting the manuscript, revised it critically for important intellectual content and gave final approval of the version to be published.

## Pre-publication history

The pre-publication history for this paper can be accessed here:


